# Editorial: The impact of the space environment on microbial growth and behavior

**DOI:** 10.3389/fmicb.2024.1390100

**Published:** 2024-03-22

**Authors:** Camilla Urbaniak, Donatella Tesei, Rob Van Houdt

**Affiliations:** ^1^NASA Jet Propulsion Laboratory, California Institute of Technology, Pasadena, CA, United States; ^2^ZIN Technologies-powered by Voyager Space, Middleburg Heights, OH, United States; ^3^University of Natural Resources and Life Sciences, Vienna, Austria; ^4^Microbiology Unit, Nuclear Medical Applications, Belgian Nuclear Research Centre (SCK CEN), Mol, Belgium

**Keywords:** space microbiology, planetary protection, microbial behavior, habitability and astrobiology, spaceflight, space environment adaptation, microorganisms

Microorganisms play an important role in life and can adapt to and survive in harsh and changing environments. Their ability to thrive in hostile conditions is reflected not only by their survival and activity in Earth's most extreme environments but also in low Earth orbit (LEO) and outer space (Olsson-Francis et al., 2018; Thombre et al., 2022). Spaceflight and the space environment present unique stressors compared to Earth (microgravity, galactic cosmic radiation, solar UV radiation, space vacuum, thermal extremes) to which microbes are exposed, but how they adapt and respond, especially in the context of deep-space exploratory missions, is still poorly understood (Tesei et al., 2022). Studies to date, though, have shown that these responses can range from being beneficial for human exploration—such as potential applications in biological life support systems (BLSS), *in situ* resource utilization (ISRU) and astronaut therapeutics (Koehle et al., 2023)—to negatively impact long duration missions (e.g., biofilm formation, increased virulence, and reduced susceptibility to antimicrobial agents), which pose risks to astronaut's health and spacecraft integrity (Wilson et al., 2007; Kim et al., 2013; Urbaniak et al., 2018). Hence, investigating the reaction of microorganisms to space conditions and the alterations in their physiology, not only helps to shed light on the molecular basis of tolerance, but also holds implications for both space exploration and astrobiology missions. This Research Topic features published articles pertaining to microbial adaption under spaceflight or simulated Mars conditions (Puig et al., Averesch et al., Blachowicz et al., Fagliarone et al., Gesztesi et al., Muñoz-Hisado et al.), life in extreme environments on Earth (Moors et al.) and planetary protection (Stott et al., Dean et al., Seto et al., Mogul et al., Kimura et al.).

The Research Topic starts off with a primary research article by Puig et al., testing the survival of genetically engineered *Escherichia coli* to simulated low earth orbit conditions. Enhanced survival to radiation, extreme temperature and low pressure was achieved through the insertion of the *Dsup* radiation resistance gene and the DNA damage repair genes, *recA* and *uvrD* (Puig et al.). DNA repair mechanisms were also shown by Fagliarone et al. to be essential for the survival of the cyanobacterium *Chroococcidiopsis* sp. CCMEE 029 in Mars cryosphere conditions, specifically the key genes *ftsZ* and *sulA*. The studies by Blachowicz et al. and Averesch et al. conducted aboard the International Space Station (ISS) provide valuable insights into the adaptability of filamentous fungi. Blachowicz et al. examined the response of *Aspergillus niger* during a 12-day growth period on the ISS, unveiling genomic, proteomic and metabolomic changes indicative of adaptive strategies (Blachowicz et al.). Averesch et al. investigated the growth of *Cladosporium sphaerospermum* on the ISS for 26 days, revealing accelerated growth and better radiation absorption compared to ground controls. Using a proteomics approach, Muñoz-Hisado et al. studied the adaptation of *Bacillus subtilis* and *Curtobacterium flacumfaciens* to growth under Martian conditions and showed that *B. subtilis* had an elevated stress response, increased catabolism and increased mobility and biofilm formation, while *C. flacumfaciens* strengthened its cell envelope to help protect the cell from the extracellular environment. The reasons for the differences observed between Mars-like conditions, LEO and Earth could be due to the diffusion limited environment of space where the change in gravity experienced by microbes is due to changes in fluid mixing responses (Gesztesi et al.). Gesztesi et al.'s calculations provide researchers with an inside look at suspension culture behavior in the diffusion-limited environment of microgravity at the scale of individual cells.

To explore factors that could influence the habitability of planetary bodies, Moors et al. conducted a study in the Dallol complex in Ethiopia. Their research revealed that specific physio-chemical parameters, such as water activity and kosmo-chaotropicity, play a crucial role in determining whether microbial life could thrive in a particular environment (Moors et al.).

As we explore the possibility of present or extant life beyond LEO, planetary protection (forward and backward contamination) becomes a key concern. As such, there is a need for appropriate sterilization and bioburden reduction methods, and for creating and testing (biological) indicators to validate sterilization. Kimura et al. compared various bioburden reduction techniques, such as dry heat, UV light, isopropyl alcohol (IPA), hydrogen peroxide (H_2_O_2_), vaporized hydrogen peroxide (VHP), and oxygen/argon plasma, and showed that dry heat is better for heat-resistant components, while VHP or plasma is recommended for non-heat-resistant components (Kimura et al.). Dean et al. investigated the use of infrared heaters to examine the survivability of heat-resistant spores, challenged the current recommended heat microbial reduction exposure of 500°C for 0.5 s and suggested a re-evaluation based on spore survival data. Stott et al. explored membrane filtration as a valid alternative to pour-plate processing as it can process larger sample volumes and reduce data variance in estimating spore bioburden on spacecraft hardware. Finally, Seto et al. explored biological indicators, specifically yeast prions, that could be used to develop, test and ultimately validate sample return mission sterilization systems, over the traditional spore-based biological indicators that are currently insufficient.

In conclusion, the research presented in this Research Topic underscores the significance of understanding fungal and bacterial biology in space environments, revealing promising pathways for leveraging their adaptability to support sustainable space exploration. However, it also emphasizes the imperative of careful consideration of planetary protection, paving the way for future experiments to advance our understanding of microbial behavior in extra-terrestrial settings.

## Author contributions

CU: Writing – original draft, Writing – review & editing. DT: Writing – original draft, Writing – review & editing. RV: Writing – original draft, Writing – review & editing.
